# Impact of gold nanoparticles shape on their cytotoxicity against human osteoblast and osteosarcoma in in vitro model. Evaluation of the safety of use and anti-cancer potential

**DOI:** 10.1007/s10856-019-6221-2

**Published:** 2019-02-12

**Authors:** Karol P. Steckiewicz, Ewelina Barcinska, Anna Malankowska, Agata Zauszkiewicz–Pawlak, Grzegorz Nowaczyk, Adriana Zaleska-Medynska, Iwona Inkielewicz-Stepniak

**Affiliations:** 1 Department of Medical Chemistry, Medical University of Gdansk, Debinki 1, 80-211 Gdansk, Poland; 2 Department of Environmental Technology, Faculty of Chemistry, University of Gdansk, Wita Stwosza 63, 80-308 Gdansk, Poland; 3 Department of Histology, Medical University of Gdansk, Debinki 1, 80-211 Gdansk, Poland; 4 NanoBioMedical Center, Adam Mickiewicz University, 61-614 Poznan, Poland

## Abstract

Due to development of nanotechnology and gold nanoparticles (AuNPs) increasing use in different areas of medicine, especially in oncology, better understanding of their potential cytotoxicity is necessary to protect patients safety. Shape and size of AuNPs is an important modulator of their cytotoxicity. Therefore, we investigated the cytotoxicity of AuNPs rods (≈39 nm length, 18 nm width), AuNPs stars (≈ 215 nm) and AuNPs spheres (≈ 6.3 nm) against human fetal osteoblast (hFOB 1.19), osteosarcoma (143B, MG63) and pancreatic duct cell (hTERT-HPNE) lines by MTT and neutral-red uptake assay. Moreover, influence of AuNPs on level of proapoptotic protein (Bax) and anti-apoptotic protein (Bcl-2) was measured by western blot. Cellular uptake of nanoparticles and ultrastructure changes were examined by transmission electron microscopy (TEM). In the present study we have proven that AuNPs stars are the most cytotoxic against human cells. We observed that cancer cells are more susceptible to AuNPs cytotoxic effect. Furthermore, AuNPs rods and AuNPs stars caused increased expression of Bax and decreased expression of Bcl-2 protein in osteosarcoma cells. We found that AuNPs penetrated through the cell membrane and caused ultrastructural changes. Our results clearly demonstrated that the cytotoxicity of AuNPs was shape-dependent. AuNPs stars with the highest anti-cancer potential were also the most cytotoxic type of tested NPs, whereas AuNPs spheres which appears to be the safest one had small anti-cancer potential.

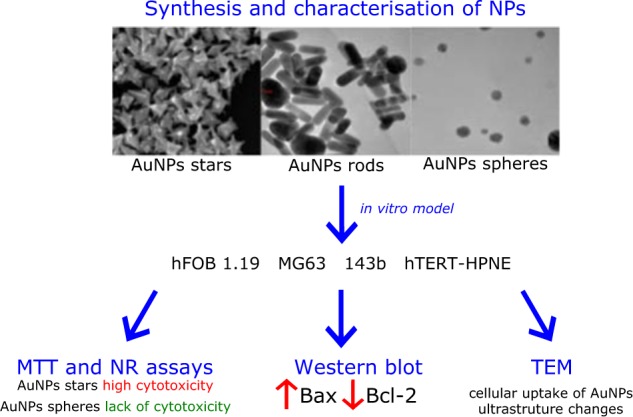

## Introduction

In 21st century nanotechnology is rapidly developing and its achievements may be used in biology and medicine. Nobel metals nanoparticles seem to be particularly interesting in biomedical application. Gold nanoparticles (AuNPs) due to small size, high surface area to volume ratio and good biocompatibility have great potential for a wide range of applications in medicine [1]. Furthermore, there are many different shapes of AuNPs, they can have one, two or even three dimension which also expand variety of potential usages [2]. It is also important that AuNPs can penetrate through biological barriers and cellular membranes. [3]. The unique properties causes that AuNPs are widely applied in diagnostic and therapy, from medical imaging [4] to bacteria and viruses detection [5, 6]. They are also component of thermal ablation [7] and cancer immunotherapy [8]. Moreover, AuNPs may be part of drug delivery systems [9]. Unfortunately, it has been shown that AuNPs can accumulate in vacuoles and induce cell death [4, 10]. In addition, AuNPs may cause increased synthesis of proapoptotoic proteins [3].

There are not enough studies which compare different shapes of AuNPs on the same cell lines using identical methodology and because of variety of potential bioapplication of AuNPs, we decided to assess the impact of shape and size of AuNPs on human cells in in vitro model. Cytotoxicity of different concentration of AuNPs rods, AuNPs stars and AuNPs spheres were tested on four cell lines: hFOB 1.19, 143B, MG63 and hTERT-HPNE. According to our knowledge it is the first study, which compares impact of shape of AuNPs on their cytotoxicity against human osteoblast, osteosarcoma and pancreatic duct cells. The main purpose of this research was to assess the cytotoxic activity against cancer cells as well as the safety of use.

## Materials and methods

### Chemical reagents

Cetyltrimethylammonium bromide (99%, CTAB), sodium borohydrate (>98%), L-ascorbic acid (99%, AA), silver nitrate (99%), tannic acid were purchased from Sigma Aldrich. Gold (III) chloride trihydrate was purchased from Alfa Aesar.

### Synthesis of AuNPs

The AuNPs spheres, rods and stars were prepared and characterized as described in our previous articles [11, 12], with some modification indicated below.

#### Au nanospheres

AuNPs spheres were obtained by mixing solution of tannic acid (3 ml, 6 × 10^−3^ M) and hot solution of HAuCl_4_ (50 ml, 1.3 × 10^−4^ M) for 1 min.

#### Au nanostars

Firstly, an aqueous solution of gold precursor (0.2 mL, 0.01 M) was added to the 0.1 M CTAB. After that 0.01 M AgNO_3_ solution and 0.1 M AA solution were added. In the next step, 20 µL of AuNPs stars solution was added. The obtained solution was kept for 20 h at 28–30 °C. The color of the solution became blue indicating the formation of AuNPs stars. The products were isolated and washing with water.

#### Au nanorods

Firstly, seed solution was obtained by stirring 0.2 M CTAB solution with 0.5 mM gold precursor and 0.6 ml of 0.01 M NaBH_4_. The solution was kept at 30 °C for 4 h. Then, AuNPs rods were prepared by mixing 5 mL CTAB, 40 mM AgNO_3_ solution, 5 mL HAuCl_4_ solution followed by the addition of 70 µL AA. The final step was the addition of 12 µL of the seed solution to the growth solution at 30 °C. The AuNPs rods were isolated and washed with water.

### Characterization of synthesized AuNPs

UV–Vis absorption spectra were obtained using a spectrophotometer Thermo Scientific Evolution 220 (Waltham, MA, USA) in the range of 200–1400 nm. The morphology and distribution size of obtained particles were observed using SEM Jeol 7001TTLS microscope operated at 12 kV and HR-TEM (ARM 200 F) operating at 200 kV. For HR-TEM sample preparation, a drop of a aqueous gold dispersion was deposited on cooper grid covered with a formal-carbon membrane. For SEM analysis aqueous solution of AuNPs was deposited on cleaned silicon wafer substrates.

### Cell culture

Cell lines were obtained from the American Type Culture Collection (ATCC). Human fetal osteoblast cell line (hFOB 1.19, ATCC CRL-11372), was cultured in 1:1 mixture of Ham’s F12 Medium and Dulbecco’s Modified Eagle’s Medium (PanBiotech, Germany), by supplemented 2.5 mM L-glutamine, 10% fetal bovine serum (FBS) and 1% of penicillin/streptomycin (P/S). Human bone osteosarcoma cell line (143B, ATCC CRL 8303) was cultured in Minimum Essential Medium (Eagle) in Earle’s BSS (PanBiotech, Germany) with 0.015 mg/mL 5-bromo-2′-deoxyuridine, 2.5 mM L-glutamine, with an addition of 10% FBS and 1% of P/S. Human osteosarcoma cell line (MG-63, ATCC CRL-1427) was cultured in Eagle’s Minimum Essential Medium (PanBiotech, Germany) supplemented by 10% FBS and 1% of P/S. hTERT-HPNE cell line (pancreatic duct cells) (ATCC CRL-4023) was cultured in medium which consist of 75% Dulbecco’s Modified Eagle’s Medium without glucose (Sigma Aldrich), 25% of M3 Base (Sigma Aldrich, USA), 5% of FBS, 1% of antibiotics, 5.5 mM D-glucose, 2 mM of L-glutamine, 1.5 g/L sodium bicarbonate and 10 ng/mL human recombinant EGF. All cells were cultured under standard conditions. All cell cultures were cultured at 37 °C in a humidified atmosphere of 5% CO_2_. Cells were maintained in 75 cm^2^ tissue culture flask. The medium was replaced every 48 h. When confluent cells were detached with trypsin-EDTA solution and subcultured into a newer flask. Subcultivation ratio was 1:4 for hFOB 1.19, 143B, and MG-63 cells and 1:8 for hTERT-HPNE cells.

### Treatments

hFOB 1.19, MG-63, 143B and hTERT-HPNE cells were treated using AuNPs in the three different shapes rods, stars, and roods for 24 h. Concentrations used in experiments were determined by preliminary studies (in range of 0.3–5 μg/mL). Each time, just before, experiment AuNPs were diluted in FBS-free media and shaken well to ensure equal dispersion of AuNPs in solution. AuNPs solutions were shaken before use to avoid agglomeration of nanoparticles. Control samples were treated with AuNPs-free, FBS-free culture media. The medium was not change during the incubation process.

### MTT viability assay

MTT assay was used to determine cell viability. Cells were seeded in 96 plates at a density of 1 × 10^4^ cell per well. After 24 h cells were treated as described in *Treatments* section. For rods, stars and spheres concentration 0.3, 0.6, 1.2, 2.5 and 5 μg/mL were examined. After 24 h incubation the media was supplemented of water-soluble tetrazolium salt [3-(4,5-dimethylthiazol-2-yl)-2,5-diphenyltetrazolium bromide (final concentration 0,45 mg/mL). Next, microplates were incubated at 37 °C in 5% CO_2_ for 2 h. After incubation media was replaced and formazan crystals were diluted in 100 μL of dimethyl sulfoxide (DMSO). After 15 min, cell viability was assessed by measuring absorbance at 540 nm (reference 630 nm) using microplate reader. Viability was determined as a percentage of control (viability of control cells was set as 100%). Absorbance values were corrected with blank NPs.

### Neutral red uptake viability assay

The assay is based on the ability of viable, uninjured cells to accumulate neutral red dye solution in lysosomes. Cells were seeded in 96 plates at a density of 1 × 10^4^ cell/well. After 24 h cells were treated as described in treatments section. For rods, stars and spheres concentration 0.3, 0.6, 1.2, 2.5 and 5 μg/mL were examined. Next, to each wall, the neutral red dye was added to final concentration of 100 μg/mL. Then, microplates were incubated at 37 °C in 5% CO_2_ for 2 h and medium was removed, cells washed with phosphate buffered saline (PBS) (NaCl 0.138 M, KCl 0.0027 M, pH = 7.4, without Ca^2+^ and Mg^2+^) and fixated with neutral red fixative solution (0.5% formaldehyde, 0.1% CaCl_2_). Subsequently, the dye was dissolved in neutral red solubilization solution (50% ethanol, 1% acetic acid) and gentle shaking for 10 min. Cell viability was assessed by measuring of absorbance at 540 nm (reference 630 nm) using microplate reader. Viability was determined as a percentage of control (viability of control cells was set as 100%). Absorbance values were corrected with blank NPs.

### Western blotting

Western blotting was used to examining the influence of AuNPs on pro and anti-apoptotic proteins levels. Briefly, MG-63 and 143B cells were treated with nanoparticles in FBS-free media as described in *Treatments* section. For AuNPs rods concentrations of NPs were 1 and 2 μg/mL, for AuNPs stars concentrations were 0.1, 0.3, 0.6, 1 μg/mL. Cells were seeded in 10 cm^2^ Petri dish. When confluence was about 80–90% cells were treated with AuNPs for 24 h and Western blotting analysis was performed according to protocol previously established by our team [13]. Before electrophoresis protein level was measured by Bradford method [14]. Rabbit polyclonal anti-Bcl-2 and anti-Bax IgG antibodies and anti-rabbit secondary antibodies were used (Santa Cruz, USA). Dilution of antibodies according to manufacturer protocol was 1:250 for Bax; 1:100 for Bcl-2 and 1:20000 for anti-rabbit secondary antibodies. β-actin was used as loading control. Immunoactive proteins level were examined by chemiluminescence (ECL) Western-blotting kit.. Proteins levels were quantified using densitometry software (ImageQuant Software, GE Healthcare, UK).

### TEM analysis

Cellular uptake of nanoparticles and ultrastructure changes were examined by transmission electron microscopy (TEM). Briefly, hTERT-HPNE cells were cultured in 10 cm^2^ Petri-dish. When 80–90% confluent cells were treated with AuNPs rods and stars in concentration 10 and 50 μg/mL as described in *Treatment* section. After 24 h of incubation cells were fixated with 2.5% glutaraldehyde in 0.1 mM sodium-cocodylate buffer. Then cells were detached and centrifuged. Cells plates was postfixaited in 2% osmium tetroxide. Next dehydration in graded solution of ethanol was applied. Cell were infiltrated with propylen dioxide, eopn mixture and pure eopn. Then cell were settled to polymerise. Prior to TEM examination at 100 kV (JEM 1200EX II, Jeol, Japan), ultra-thin section (Reichert OmU3 ultramicrotome, Austria), were contrasted by uranyl acetate and lead citrate.

### Statistical analysis

All statistical analysis was performed with GraphPad Prism 5 software (GraphPad Software, Inc, USA). All data on graphs are presented as the mean ± standard error of 3-4 independent experiments. Statistical analysis was determined by one-way analysis of variance (ANOVA) and Tukey’s posthoc test. The IC_50_ was calculated by analyzing of non-linear regression log(inhibitor) vs normalized response.

## Results and discussion

### Morphology of AuNPs

**Fig. 1:**
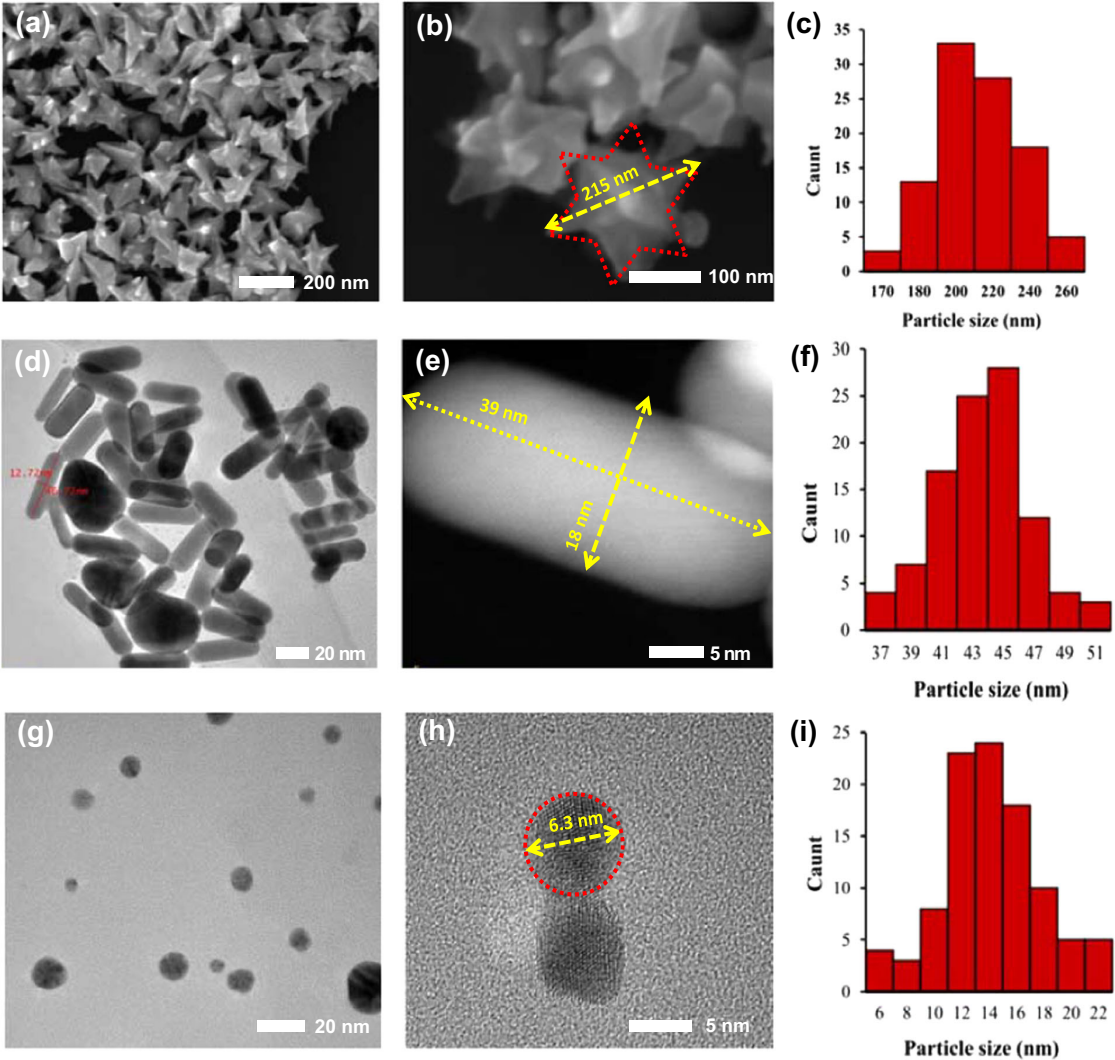
Morphology (TEM/SEM images) and average size distribution of **a**–**c** AuNPs stars, **d**–**f** AuNPs rods, **g**–**i** AuNPs spheres

The morphology of prepared samples was studied by SEM and TEM microscopy. The average gold size was calculated from average size of 100 AuNPs using ImageJ Analysis Software. As clearly shown (Fig. 1a–c), the *AuNPs* stars have well-developed with the tip-to-tip diameter in range 170–260 nm with various numbers of tips. The major fraction of AuNPs stars appears with an average size of ~ 200 nm. SEM analysis also showed that all of AuNPs stars particles have a branched structure. The fractions in diameter about 170 nm and 260 nm represented a small part in the test sample. The AuNPs rods with narrow size distribution of ~ 45 nm in length and ~16 nm diameter are shown in Fig. 1d–f. The major fraction of AuNPs rods appears with an average length size ~ 45 nm. Moreover, observation at high magnification shows that the surface of the AuNPs rods is smooth. The TEM results also confirmed that small fraction of the formed particles have a spherical shape. Nikoobakht *et al*. showed that formation of a large fraction of spherical particles can be overcome by use of a (CTAB)-capped seed instead of a citrate-capped one [15]. After reduction of gold precursor by tannic acid, the gold AuNPs spheres with diameters in the range from 6 to approximately 22 nm were formed (Fig. 1g–i). AuNPs spheres were rather uniform in shape. The major fraction of AuNPs spheres appears with an average size equaled to 14 nm.

### UV-Vis properties of gold nanoparticles

**Fig. 2:**
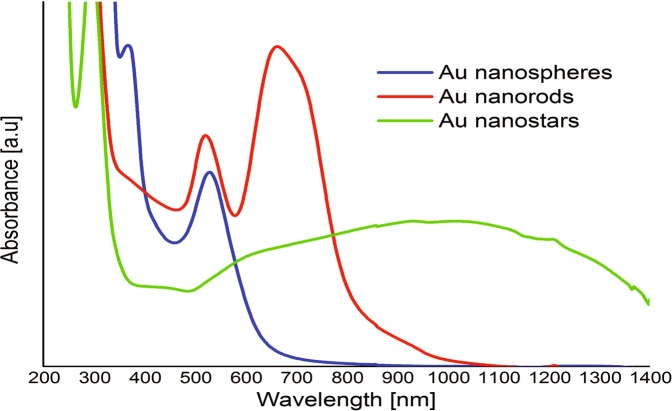
UV-Vis spectra of AuNPs spheres, AuNPs rods and AuNPs stars

The UV-Vis properties of prepared gold nanoparticles were characterized by UV–Vis spectroscopy in range 200–1400 nm (Fig. 2). The AuNPs exhibit a distinct optical feature commonly referred to as localized surface plasmon resonance (LSPR). The position and intensity of the LSPR band depends on the size and surface morphology of gold particles (a–b). For AuNPs spheres, the plasmon peak shifts to higher wavelengths with increasing particle size, from the visible to the IR light [16]. The absorption band at 530 nm was observed for AuNPs spheres and this peak position comes from small particles, which is also confirmed by TEM results. According to the literature, the one plasmon band around 527 nm is corresponding to the spherical gold with size about 20 nm [17]. For AuNPs stars a plasmon band ranging from 500 to 1400 nm was observed. According to the literature, the absorption peak in the IR region depends on the number of tips of gold nanoparticles [18]. It is know that the shape of the branches and their each other interaction of AuNPs stars determine the absorption ranges [16]. For AuNPs rods, typically two plasmon resonances are observed. The transverse and longitudinal LSPR extinction peaks located around 520 and 680 nm respectively, was observed for AuNPs rods prepared using seed-mediated synthesis. Appearance of transverse and longitudinal plasmon resonances is evident of the formation of AuNPs rods. Further, the presence of two characteristic peaks suggests that the sample was homogenous.

### Determination of cell viability

**Fig. 3:**
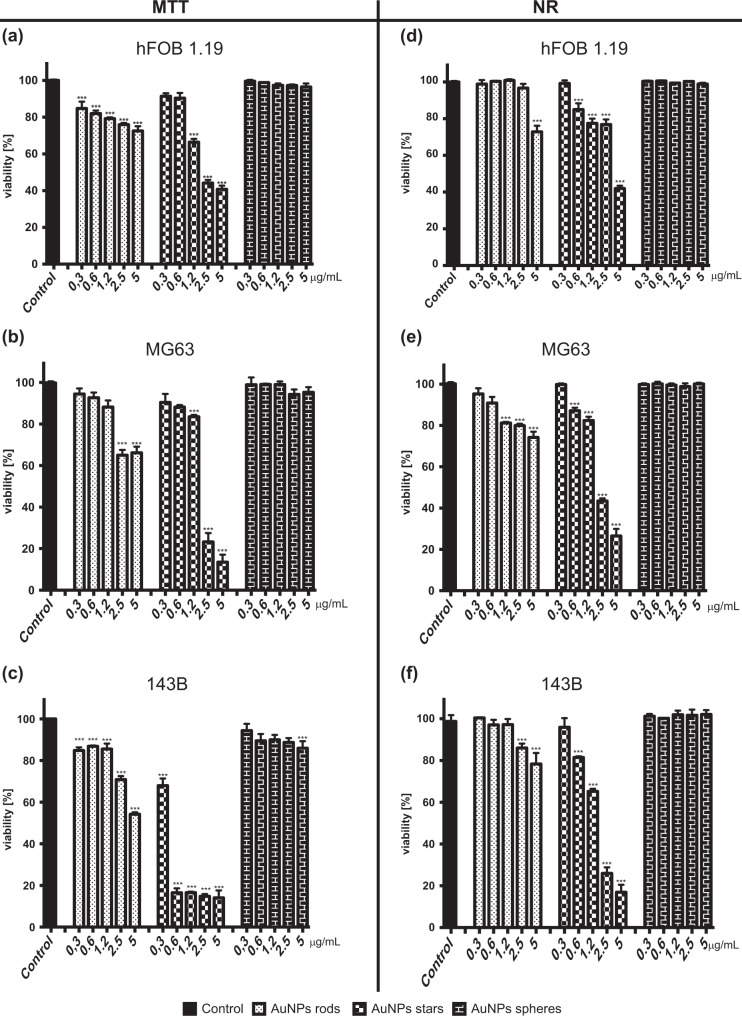
Different shapes of AuNPs decreased cell viability in a concentration-dependent manner. Viability, measured by MTT test, of **a** hFOB1.19 cells, **b** MG-63, **c** 143B cells exposed to different shapes of AuNPs after 24 h. Viability, measured by NR test, of **d** hFOB1.19 cells, **e** MG-63, **f** 143B cells exposed to different shapes of AuNPs after 24 h. Data are presented as mean ± SD. *p < 0.05, **p < 0.01, ***, *p* < 0.001

Analysis of MTT assay and NR assay results have shown that shape and concentration of nanoparticles has an impact on their cytotoxicity (Fig. 3)

The highest impact on cells survival had AuNPs stars and decreased cells viability in a concentration-dependent manner. MTT assay has shown that AuNPs stars significantly decreased the viability of hFOB 1.19 in concentration range 1.2–5 μg/mL, MG-63 in concentration 1.2–5 μg/mL, and 143B in concentration range 0.3–5 μg/mL, whereas NR assay did not prove the cytotoxic effect of AuNPs stars in the lowest concentration (0.3 μg/mL). The most susceptible to cytotoxic effect of AuNPs stars were 143B cells. For the high concentration of AuNPs stars (2.5 and 5 μg/mL) hFOB 1.19 cells were the most resistant one. After exposure to the low concentrations of AuNPs stars (0.3 and 0.6 μg/mL) hFOB 1.19 and MG-63 cells had similar viability.

In MTT assay AuNPs rods significantly decreased the viability of hFOB 1.19, MG-63 and 143B cells. However, other assay (NR assay) has proven that hFOB 1.19 are resistant to cytotoxic effect of AuNP rods in concentration between 0.3–2.5 μg/mL, MG-63 in concentration range 0.3–0.6 μg/mL and 143B cells were resistant to AuNPs rods in concentration range of 0.3–1.2 μg/mL.

AuNPs spheres exerted the smallest cytotoxic effect compared to other analysed nanoparticles. AuNPs spheres did not decrease the viability of hFOB1.19 and MG-63 cells, examined by MTT assay. AuNPs spheres, in concentration 5 μg/mL, decreased the viability of 143B cells but the effect was lower in comparison to other shapes. In NR assay AuNPs spheres did not have any statistically significant effect on the viability of hFOB1.19, MG63 and 143B cells in the analysed range of concentration. Non-linear regression analysis: log(inhibitor) vs. normalized response has been performed to calculate log IC_50_ values (online resource 1). IC_50_ values for AuNPs stars are presented in Table 1. In order to show higher cytotoxicity of AuNPs stars against cancer cell lines compered to non-cancer cells.

**Table 1:** I_C50_ for AuNPs stars

	HFOB1.19	MG-63	143B
**MTT ASSAY**	1.241 μg/mL	1.760 μg/mL	0.4266 μg/mL
**NR ASSAY**	3.961 μg/mL	1.841 μg/mL	1.396 μg/mL

### Protein level of Bax and Bcl-2

**Fig. 4:**
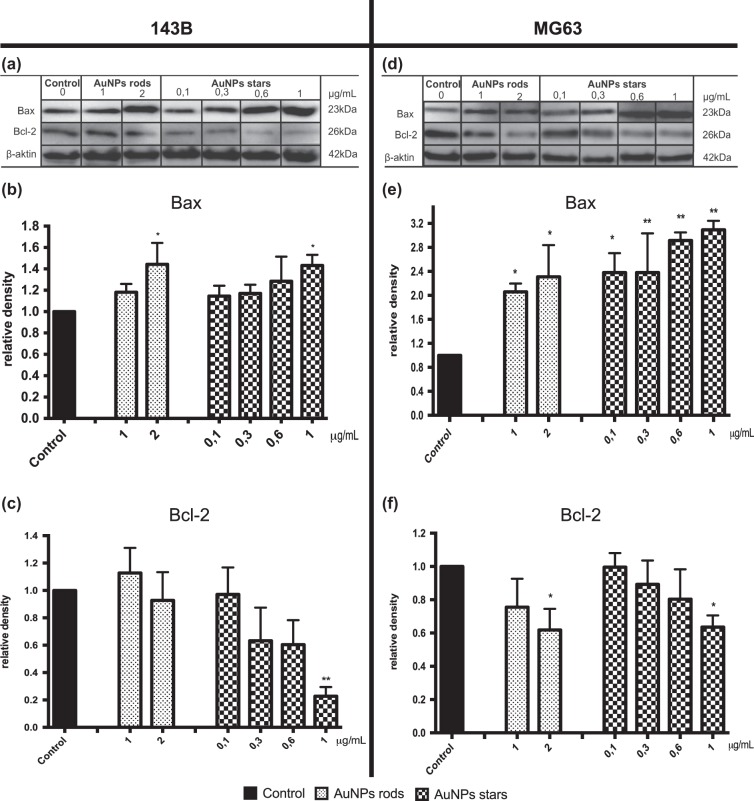
Western-blot analysis of apoptosis-related protein level in 143B and MG-63 cells after 24 h of incubation with AgNPs. Representative Western blot analysis of Bax and Bcl-2 in **a**) 143Bcells and **d** MG-63. Quantitive analysis of **b**, **e** Bax and **c**, **f** Bcl-2 proteins in 143B and MG-63 cells, respectively. Data are presented as mean ± SD. *p < 0.05, **p < 0.01

We determined the impact of AuNPs rods and AuNPs stars on apoptosis-related protein. Level of proapoptotic protein (Bax) and anti-apoptotic protein (Bcl-2) in MG-63 and 143B cells was demonstrated (Fig. 4). Due to lack cytotoxicity showed in NR assay and small cytotoxic effect (only in concentration 5 μg/mL) against 143B cells only showed by MTT assay, we did not determine the influence of AuNPs spheres on the level of protein, which are crucial regulators of cell death. AuNPs rods significantly increased the protein level of Bax in both cell lines, however, decreased the level of Bcl-2 was observed only in MG-63 cells. AuNPs stars significantly increased level of Bax and decreased level of Bcl-2 in all tested cell lines. For MG-63 cells AuNPs stars increased level of Bax protein in concentration between 0.1–1 μg/mL and decreased level of Bcl-2 protein in concentration of 1 μg/mL. AuNPs rods in concentration between 1–2 μg/mL increased level of Bax protein and in concentration of 2 μg/mL decreased level of Bcl-2 protein in MG-63 cells. AuNPs stars in concentration of 1 μg/mL increased level of Bax protein and decreased level of Bcl-2 protein on 143B cells. In 143B cells AuNPs rods in concentration of 2 μg/mL increased level of Bax protein, however AuNPs rods in tested range of concentration did not, statistically significant, influence level of Bcl-2 protein in 143B cells.

### TEM analysis

**Fig. 5:**
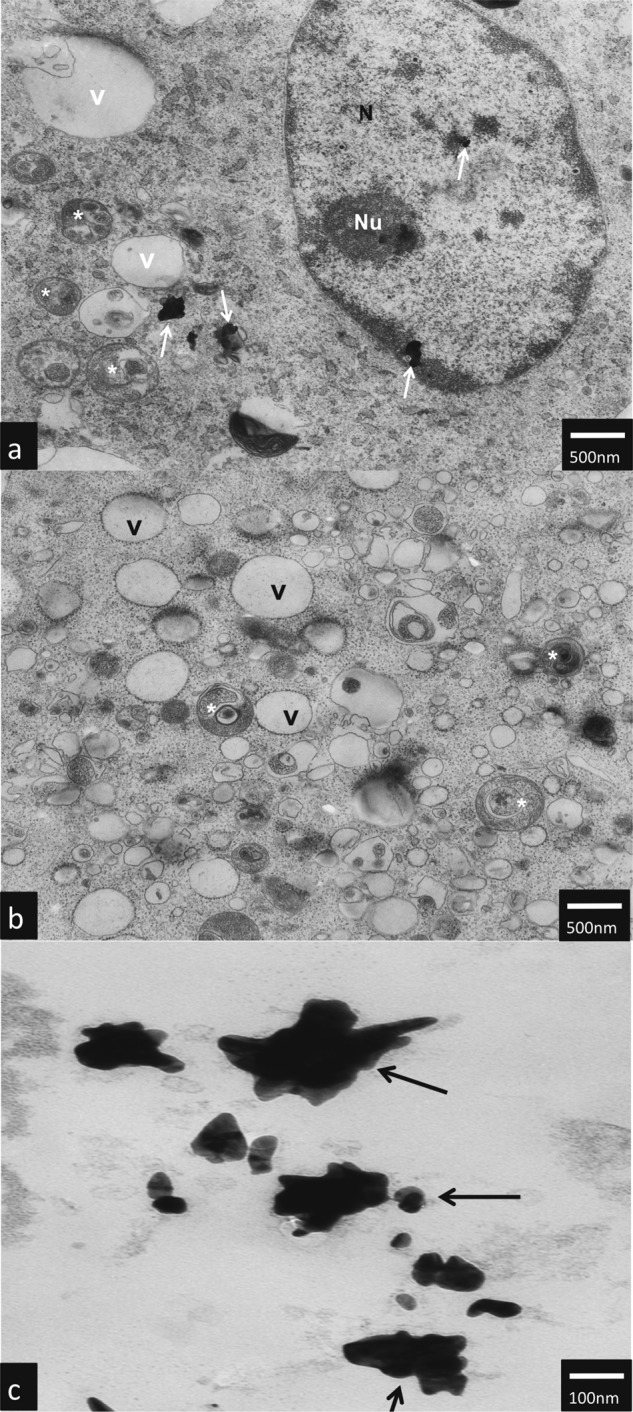
Ultrastructure changes in hTERT-HPNE cells after 24 h incubation with 10 μg/mL AuNPs stars. AuNPs stars are indicated by arrows, N nulceus, NU nucleous, V vaculoes, *-authophagic vacuoles. The scale bar is present on the left side of each picture

**Fig. 6:**
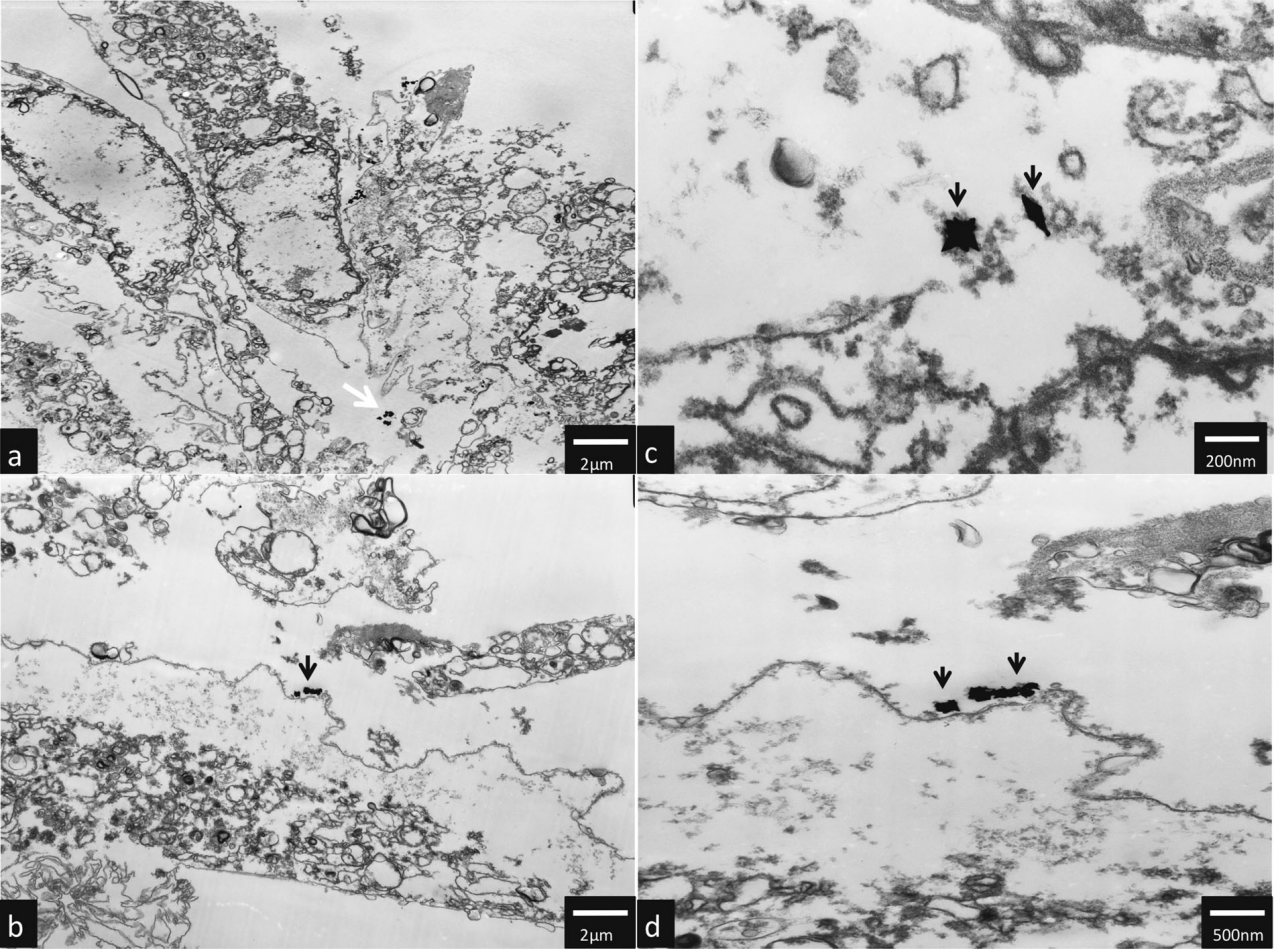
Ultrastructure changes in hTERT-HPNE cells after 24 h incubation with 50 μg/mL AuNPs stars. AuNPs stars are indicated by arrows. The scale bar is present on the left side of each picture

**Fig. 7:**
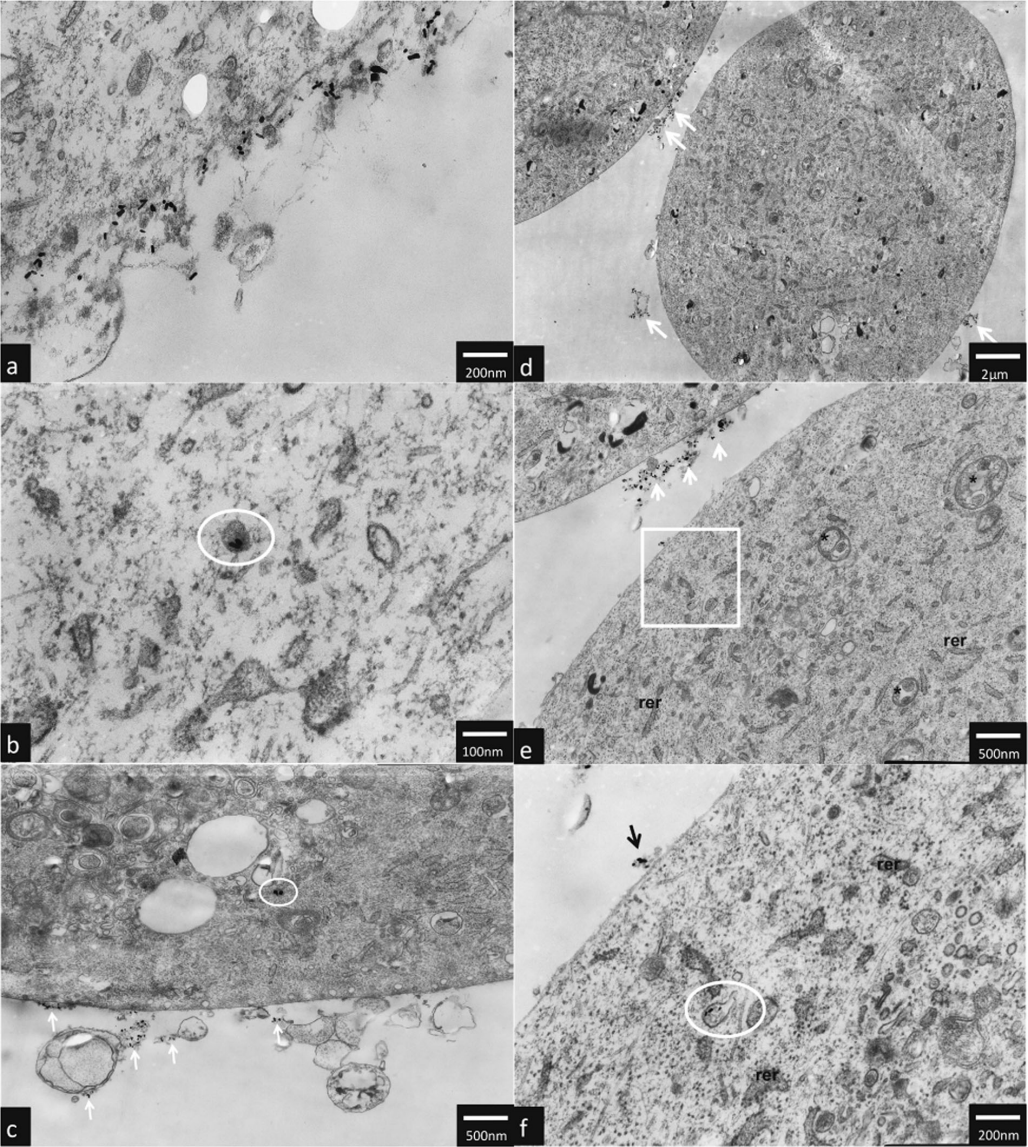
Ultrastructure changes in hTERT-HPNE cells after 24 h incubation with 10 μg/mL AuNPs rods. AuNPs rods are indicated by arrows. Endosomes are circled, RER rough endoplasmatic reticulum, * authophagosomes. The scale bar is present on the left side of each picture

**Fig. 8:**
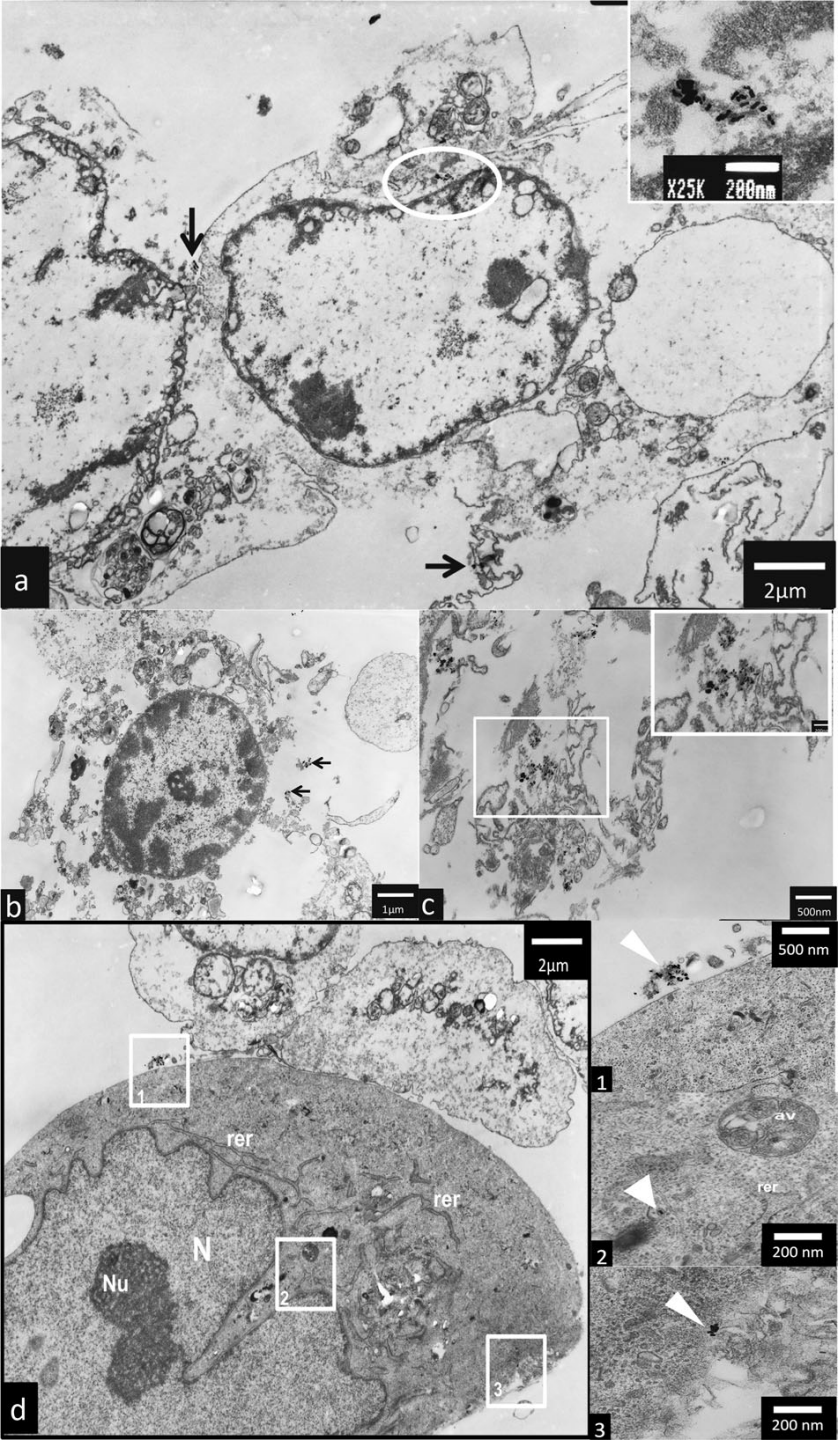
Ultrastructure changes in hTERT-HPNE cells after 24 h incubation with 50 μg/mL AuNPs rods. AuNPs rods are indicated by arrows. AuNPs rods were founded near the nuclear membrane (boxed), RER rough endoplasmatic reticulum, AV authophagic vacuoles. The scale bar is present on the left side or at the bottom of each picture

TEM analysis have shown that AuNPs rods and AuNPs stars can be internalized by hTERT-HPNE cells and caused ultrastructure changes. AuNPs stars in concentration of 10 μg/mL were internalized in the cytoplasm (Fig. 5c as well as in the nucleus of the cell (Fig. 5a). Additionally, we observed intensive vacuolization of the cytoplasm, and numerous autophagic vacuoles (Fig. 5a, b). In hTERT-HPNE cells after treatment with high (50 μg/mL) concentrations of AuNPs stars, we have observed major impairment of the cells such as cell membrane rupture, cytoplasm vacuolization and general degeneration. AuNPs stars were present within cell debris (Fig. 6a–d). AuNPs rods in concentration of 10 μg/mL were found outside the cell along the cell membrane as well as internalized inside a small dense vesicles (endosomes) (Fig. 7a–f). Morphology of the cells treated with rods of AuNPs revealed normal/unchanged rough endoplasmic reticulum and numerous autophagosomes. After treatment with higher concentrations of AuNPs rods (50 μg/mL) cells underwent major degeneration. AuNPs rods have been observed along the cell membranes or cell debris (Fig. 8a–c). Some internalized of AuNPs rods have been found near the nuclear membrane (Fig. 8a). Despite the fact that the majority of cells have been seriously damaged, some cells remained normal. However, internalized AuNPs rods have been found in the cell perikaryon (Fig. 8d). The cell showed prominent rough endoplasmic reticulum as well as autophagic vacuole.

## Discussion

The aim of our research was to determine the dependence of shape and concentration on the cytotoxicity of AuNPs against human fetal osteoblast and osteosarcoma cells. We also were focused on determining the type of programmed cell death induced by AuNPs We found that, AuNPs exerted their cytotoxic effect in a shape- and concentration-dependent manner. AuNPs stars were the most cytotoxic, whereas AuNPs spheres were the less cytotoxic ones. NR assay has shown that hFOB1.19 cells were the most resistant and 143B cells were the most susceptible to all examined AuNPs. In general, the NR assay has shown the higher viability of the cells than MTT test in the same condition. Our study has proven that both cytotoxicity of AuNPs and anti-cancer potential is shape-dependent. Thus, it should be taken in concern when designing NPs for biomedical usage, in order to increase safety of NPs application.

Osteosarcoma is highly metastatic mesenchymal cells carcinoma [19]. It is the third most common cancer in youth, so osteosarcoma is substantial epidemiological problem [19]. Typical treatment of this neoplasm consists of surgery, chemotherapy and radiotherapy, so therapy is crippling and the outcome is poor [19]. There is strong requirement for improved treatment, it has been demonstarted that nanoparticles may be interesting alternative for the ‘classical’ treatment [20]. Rahim et al., demonstrated that 24.3 nm spherical AuNPs capped with glication products (Schiff’s base, Heyns products, fructosylamine etc.) inhibit growth of SaoS-2 (human osteosarcoma cell line) [21]. Similarly, Cebrain et al. have shown that 6 nm poly(ethylenimine) coated AuNPs decreased viability of SaoS-2 cells [22]. However, there was no study compering cytotoxicity of different shapes of AuNPs against osteosarcoma cells. In our study, we decided to use four cell lines hFOB1.19, hTERT-HPNE, MG-63 and 143B, because it has been proven that response to AuNPs exposure is very cell line dependent. [23]. We have chosen two osteosarcoma cell lines (MG-63 and 143B) because of their different characteristics. 143B cells proliferate and migrate more intensively than MG-63 cells, also 143B cells have higher tumorigenicity and colony forming ability [24, 25]. Taken together,143B cell line is more aggressive one. We used non-transformed and cancer cell lines, as studies suggest that cancer cells are more vulnerable to xenobiotics, due to faster and bigger uptake caused by hyper metabolism [26]. Non-transformed cells (hFOB1.19 and hTERT-HPNE) were used to assess the safety of potential in vivo application of AuNPs of different shapes. In other studies hTERT-HPNE cells were used as a comparison for selective cytotoxicity of tested compound against cancer cells. For example, Ramalho et al. compared the cytotoxicity of functionalized nanoparticles (PLGA-AuNPs) with potential anti-cancer activity against A549 cells (human lung carcinoma) and hTERT-HPNE [27]. Wada et al., also compered cytotoxicity of tested compunds on different cell lines CHO-K1 (chinese hamster ovary), HeLa (cervix cancer cells) and SH-SY5Y (neuroblast cells) [28].

### Cytotoxicity of AuNPs

In order to provide the most reliable results, we decided to use two test: MTT and NR. NR assay is based on the ability of viable cells to uptake and accumulate dye in lysosomes and measured cellular membrane integrity [29]. MTT assay measured the activity of cellular NAD(P)H dependent oxidoreductase [29]. Decreased cell viability measured by MTT may indicate the cells underwent apoptosis [29, 30]. Because of different characteristic of both assays, they do not give equal results [31]. MTT test, as well as NR assay, are commonly used to assess the cytotoxicity of nanoparticles [32–34].

Recently, several groups have focused their attention on the cytotoxic activity of AuNPs [35, 36]. Size, shape, concentration, incubation time, synthesis method, surface functionalization, type of cells are thought to have an impact of cytotoxicity of AuNPs [37]. It has been proven that AuNPs can reduce the viability of human hepatocellular carcinoma [38] and human breast adenocarcinoma [39]. On the other hand, Gannon et al. have found that AuNPs in concentration between 1 and 67 μM/L are not cytotoxic to Hep3B (hepatocellular carcinoma) and Panc-1 (pancreatic epithelioid carcinoma) cells [40]. Patra et al., demonstrated that 33 nm AuNPs were toxic to human carcinoma lung cell line (A549 cells), and did not decrease viability of human hepatocellular carcinoma cells (Hepg-2) cells [41]. In other study it has been shown that 10 and 50 nm citrate coated AuNPs were not toxic to embryonic fibroblast [42].

Size of nanoparticles is important if considering their cytotoxicity. Generally, it seems that the larger the size of nanoparticles is the less cytotoxic they exerted [43]. Indeed, Coradeghini et al. have proven that 5 nm AuNPs were more cytotoxic in comparison to 15 nm AuNPs on Balb/3T3 (mouse fibroblast) cells. [44]. Similarly, Senut et al. have proven that 1.5 nm AuNPs are more cytotoxic to hESC (human embryonic stem cells) cells than 4 and 15 nm AuNPs [45]. However, Vetten et al., demonstrated that 20 nm AuNPs were more cytotoxic than 14 nm on BEAS-2B cells [46].

Although extensive knowledge about AuNPs cytotoxicity there is only few publication which has taken in concern shape of NPs as an important modulator of cytotoxicity. Our results suggest that AuNPs exerted their cytotoxicity mainly by influencing mitochondria functioning (MTT assay). However, the decreased viability of cells in NR assay suggested that NPs affected integrity of cellular membranes. It has been found that AuNPs rods exerted cytotoxic effect against canine MDCK (canine kidney epithelial cells) and HEp-2 (human HeLa contaminant epithelial cells) cell lines in a concentration-dependent manner (viability of cells was measured by MTT assay) [47]. In in vitro study, Favi et. al examined the impact of AuNPs rods (length 534 ± 38 nm, width 65 ± 8 nm) on the viability of human dermal fibroblast. They observed that viability of the cells measured by MTS assay was decreased by 10–15% by AuNPs rods at concentration of 400 μg/mL [48]. In our study, AuNPs rods in concentration of 5 μg/mL decreased MG-63 cells viability (measured by MTT assay) by approximately 34% and 143B cells by 46%. There are significant differences between our results and results presented by Favi et al. Firstly, they examined AuNPs rods in bigger size, and it has been proven that the bigger nanoparticles are the smaller effect on cells viability they have [43]. Furthermore, MTT and MTS test give similar but not equal results [49]. Other studies have proven that AuNPs rods decreased the viability of A549 cells (human lung adenocarcinoma cells) in a concentration-dependent manner. Further, it has been observed, consistent with our results, that AuNPs rods (length 40 nm) are more cytotoxic than AuNPs spheres [47]. In several studies, it has demonstrated that AuNPs spheres did not have cytotoxic activity [43, 50].

### AuNPs-induced programmed cell death

Choudhury et al., observed decreased level of Bcl-2 (anti-apoptotic protein) and increased level of Bax (proapoptotic protein) in A549 cells after incubation with 40 nm AuNPs [51]. Selim et al., have reported that AuNPs may increase mRNA level of proapoptotic protein Bax, and decreased the level of a protein Bcl-2 in MCF-7 cells (human mammary adenocarcinoma) [39]. Similar results were presented for Hepg-2 cells incubated with 14.5 nm spherical AuNPs [52]. AuNPs rods are thought to induce apoptosis [30, 47]. Furthermore, Chueh et al., have proven that AuNPs rods (length 10–40 nm) induce apoptosis and autophagy in NIH3T3 cells (mouse fibroblast) [23]. Ding et al., have observed that spherical AuNPs (5, 13 nm) caused autophagy in HK2 cells (human renal proximal tubular cells) [53]. Tang et al., have ascertained that AuNPs rods (width 23–26 nm, length 35–58 nm) may cause necrosis of A549 cells. Furthermore, necrotic cells ratio increases in presence of high concentration of AuNPs rods (in concentration > 10 μg/mL) [54]. Our results suggest that AuNPs rods and AuNPs stars may induced apoptosis in MG-63 and 143B cells, which is similar to observations made by several other authors [30, 47, 52].

### Cellular uptake and ultrastructure changes

AuNPs may be internalized into cells and caused ultrastructural changes. Generally, molecules with positively charger surfaces have higher uptake ratio but lower intracellular stability in comparison to neutral or negatively charged molecules [55]. Furthermore, size of nanoparticles influence effectivity of their internalization [56]. There are two main mechanisms of AuNPs internalisation by membrane-bound vesicles [35] and endosomes [57]. Receptor-mediated endocytosis and fluid-phase endocytosis are the additional way of AuNPs internalisation [58]. Mironava et al., have demonstrated that way of AuNPs internalisation depends on diameter of AuNPs [10]. 45 nm AuNPs penetrate into human dermal fibroblast by clathrin-mediated endocytosis, while for 13 nm AuNPs phagocytosis is main way of internalisation [59]. It has been proven that AuNPs rods may be internalised by endosomes and vesicular bodies into human dermal fibroblasts (AuNPs rods: width 11.2–12.8 nm length 58–62 nm), colon adenocarcinoma and other cells [60, 61]. Other studies have shown that AuNPs are internalised by phagocytosis in A549 (AuNPs rods: width 23–26 nm, length: 35–58 nm) and HBL-100 cells (AuNPs spheres 20–45 nm) [54, 58]. Furthermore, AuNPs can be found in the cytosol, lysosomes and perinuclear region either in form of aggregates or single NPs [53, 54, 58]. Exposition of A549 cells to AuNPs rods (width 23–26 nm, length 35–58 nm) caused an increased number of lysosomes and swallowing of mitochondria [54]. The nucleus of A549 cells was not affected by AuNPs rods [54]. The data about uptake and cytotoxicity of AuNPs are inconsistent. Connor et al., have proven that AuNPs spheres may be taken up by K562 (chronic myelogenous leukemia) cells, but they are not cytotoxic [62]. Gannon et al. proved that AuNPs can be internalized by Panc-1 cells, however, TEM analysis has shown that AuNPs do not harm cellular organelles [40].

To the our knowledge this is the first study to compare shape- and size-dependent cytotoxic against human fetal osteoblast and osteosarcoma cells including the type of cell death and ultrastructure alterations caused by AuNPs.

## Conclusions

In the present study we demonstrated that cytotoxicity of AuNPs is depended on the shape. We found that AuNPs stars are the most cytotoxic ones. Furthermore, we observed that cancer cells are more susceptible to AuNPs. For AuNPs in all investigated shapes, IC_50_ values were the lowest for 143B cell line in comparison to hFOB 1.19 and MG-63 cell lines. We proved that AuNPs induced apoptosis in human osteosarcoma cells, both in 143B and MG-63. Moreover, AuNPs penetrated through the cell membrane and caused ultrastructural changes. Our study has proven that shape is important modulator of AuNPs cytotoxicity. Both anti-cancer potential and cytotoxicity of AuNPs is shape-dependent. It should be concerned in order to provide the highest efficiency with the highest safety of AuNPs application.

## Supplementary information

Supplementary Information
