# Supplementary material for: Impact of gold nanoparticles shape on their cytotoxicity against human osteoblast and osteosarcoma in in vitro model. Evaluation of the safety of use and anti-cancer potential
Source: J Mater Sci Mater Med. 2019 Feb 12;30(2):22. doi: 10.1007/s10856-019-6221-2 (PMC6373298; doi:10.1007/s10856-019-6221-2)
Supplement: Supplementary file 1 — Supplementary Information [file 10856_2019_6221_MOESM1_ESM.pdf]

Impact of gold nanoparticles shape on their cytotoxicity against human osteoblast and osteosarcoma in *in vitro* model. Evaluation of the safety of use and anti-cancer potential.

Karol P. Steckiewicz[1], Ewelina Barcińska[1], Anna Malankowska[2], Agata Zauszkiewicz – Pawlak[4], Grzegorz Nowaczyk[3], Adriana Zaleska-Medynska[2], Iwona Inkielewicz-Stępniak[1]

[1]Department of Medical Chemistry, Medical University of Gdańsk, Dębinki 1, 80-211 Gdansk, Poland

[2]Department of Environmental Technology, Faculty of Chemistry, University of Gdansk, Wita Stwosza 63, 80-308 Gdansk, Poland

[3]NanoBioMedical Center, Adam Mickiewicz University, 61-614 Poznan, Poland

[4]Department of Histology, Medical University of Gdańsk, Dębinki 1, 80-211 Gdansk, Poland

Corresponding author: Iwona Inkielewicz-Stepniak, iinkiel@gumed.edu.pl, *tel. +48* 58 349 1450

Electronic Supplementary Material

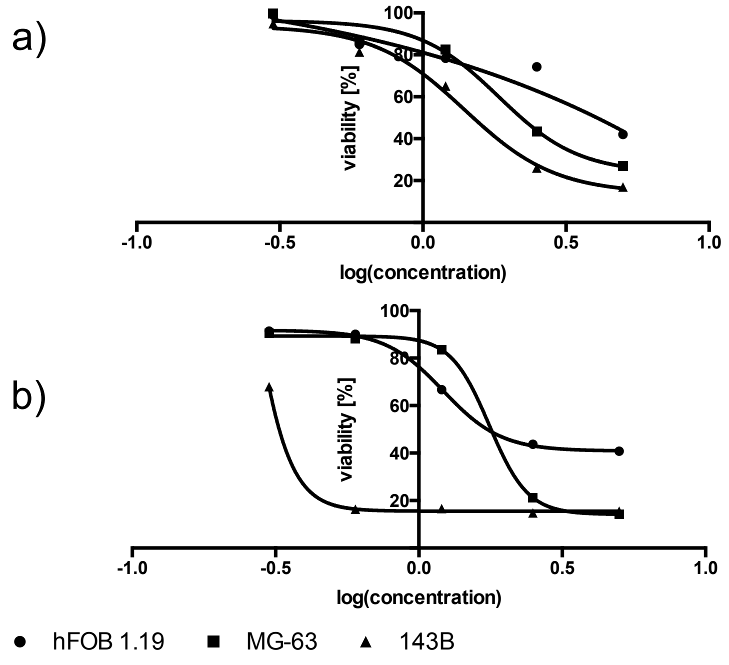

Fig. Log(concentration) vs. cell viability plots. Non-linear regression analysis: log(inhibitor) vs. normalized response has been performed to calculate log IC50 values.Calculation were made for three cell lines (hFOB1.19, MG-63 and 143B), using (a) MTT and (b) NR assay.The obtained data are reported as the mean ± SD for triplicate determination of 3 separate experiments
